# Synthesis of Magnetic Wood Fiber Board and Corresponding Multi-Layer Magnetic Composite Board, with Electromagnetic Wave Absorbing Properties

**DOI:** 10.3390/nano8060441

**Published:** 2018-06-16

**Authors:** Zhichao Lou, Yao Zhang, Ming Zhou, He Han, Jiabin Cai, Lintian Yang, Chenglong Yuan, Yanjun Li

**Affiliations:** College of Materials Science and Engineering, Nanjing Forestry University, Nanjing 210037, China; seubmelzc2005@163.com (Y.Z.); zzzzm12123@163.com (M.Z.); 18805166615@163.com (H.H.); nldfloor@163.com (J.C.); 13771214742@163.com (L.Y.); 18331276712@163.com (C.Y.)

**Keywords:** magnetic board, electromagnetic wave absorption, co-precipitation, hot-pressing

## Abstract

With the rapid growth in the use of wireless electronic devices, society urgently needs electromagnetic wave (EMW) absorbing material with light weight, thin thickness, wide effective absorbing band width, and strong absorption capacity. Herein, the multi-layer magnetic composite boards are fabricated by hot-pressing magnetic fiber boards and normal veneer layer-by-layer. The magnetic fibers obtained using in-situ chemical co-precipitation are used to fabricate magnetic fiber board by hot-pressing. The magnetic wave absorbing capacities of the magnetic fiber boards obtained with 72 h impregnation time exhibit strongest adsorption capacities of −51.01 dB with a thickness of 3.00 mm. It is proved that this outstanding EMW absorption property is due to the strongest dielectric loss, the optimal magnetic loss, and the dipole relaxation polarization. Meanwhile, the EMW absorbing capacities of the corresponding multi-layer composite magnetic board increases from −14.14 dB (3-layer) to −60.16 dB (7-layer). This is due to the generated multi-interfaces between magnetic fiber board and natural wood veneer in the EMW propagation direction, which significantly benefit multireflection and attenuation of the incident waves. The results obtained in this work indicate that natural wood fibers are of great potential in the fabrication of magnetic multi-layer boards treated as EMW absorbers via a low cost, green, and scalable method.

## 1. Introduction

With the rapid growth in the use of wireless electronic devices such as mobile phones, local area networks, and household robots, people’s daily life and production are increasingly affected by electromagnetic waves generated by electromagnetic systems. As a result, the society urgently needs electromagnetic wave (EMW) absorbing materials with light weight, thin thickness, wide effective absorbing band width, and strong absorption capacity [1,2,3,4]. These absorbers include polymer composites with low conductive nanoparticles [5,6], polymer composites with metallic nanoparticles [7], composites containing glass coated microwires [8,9], thin film based composites [10,11], magnetic cellulose [12], and magnetic wood [4,13].

Wood is a biomass resource, which is renewable and naturally degradable. It is widely used in furniture and decoration, and the fabrication of wood-based EMW absorption materials has become one of the hot spots to further increase the added value of boards [14]. Many research groups have fabricated a series of metal-biomass composite boards with good EMW absorption properties by chemical plating [15], or using a laminated method [16]. However, these metal-biomass composite boards have limitations in application, which are mainly reflected in the following aspects: 1. large densities due to the appearance of heavy metal components, losing the excellent strength-to-weight ratio of natural wood; 2. poor properties in weather resistance and chemical resistance; 3. poor fabricability; 4. poor adjustability of the electromagnetic wave absorption properties (e.g., maximum absorption value and the corresponding matching frequency) according to different application requirements. To solve these problems, the Oka group firstly proposed magnetic wood, which is basically the combination of wood with a magnetic fluid or powder [14,17,18]. This magnetic wood has been proven to possess strong magnetic characteristics and a wave-absorption function. In addition, magnetic wood also offers a wood texture, low specific gravity, and is very easy to process, which is expected as an indoor wave absorber to extend the wood supply, improve the value of wooden products, and preserve natural resources from over-exploitation [4,19,20].

At present, there are three typical manufacturing methods for magnetic wood, which are named as the impregnated [17], powder [18], and coating types [21]. Although through these methods, the researchers can obtain magnetic wood with excellent EMW absorbing properties, there are still some disadvantages which restrict their application. For example, some of these processes involve finished products of magnetic particles, resulting in the corresponding manufacturing cost being greatly dependent on the upstream enterprises. In addition, the inter-particle dipolar forces easily lead to aggregation of magnetic particles, which worsens during the fabrication processes, affecting the uniformity of EMW absorption performance in different parts of the magnetic wood. Some groups have impregnated the pretreated wood in a mixed solution of Fe^3+^ and Fe^2+^, followed by impregnation of ammonia solution [4,20,22]. By doing this, Fe_3_O_4_ nanoparticles were synthesized via in situ co-precipitation chemical reaction in the wood, manufacturing the magnetic wood. However, the fabrication of magnetic fiber board via in situ chemical co-precipitation and laminated hot-pressing, along with the investigation of the corresponding EMW absorbing properties, is rarely studied.

In this paper, the multi-layer magnetic wood was fabricated by hot-pressing the magnetic fiber board and the normal veneer layer-by-layer. The obtained magnetic fibers, by in situ chemical co-precipitation, were used to fabricate magnetic fiber board by hot-pressing. SEM, XRD, FT-IR, VSM and the network vector analyzer were used to investigate the morphology, composition, magnetic and electromagnetic wave absorption properties of the synthesized samples. The results showed that the magnetic properties of magnetic fibers, as well as their EMW absorbing properties, varied according to the impregnation time of the iron mixed solution. Furthermore, we studied the influence of the number of the contained magnetic fiber boards in the multi-layer magnetic board on the shielding performance. All the results confirmed that the obtained magnetic fiber board in our work has an excellent and tunable absorbing property, which was due to the optimal impedance matching, strongest dielectric loss, optimal magnetic loss, interface polarization between biomass-Fe_3_O_4_, and interconnected conductive network for electron hopping and migrating.

## 2. Materials and Methods

### 2.1. Experimental Materials

*Populus* spp. fiber with length of 1–2 mm and veneer with thickness of around 2 mm were obtained from Jinmu Arts & Crafts Co., Ltd., Fuyang, Anhui province in China. The initial moisture content of both the fiber and veneer was less than 10%. FeCl_3_·6H_2_O, FeCl_2_·4H_2_O and NH_3_·H_2_O (25%) was purchased from Sigma-aldrich company (Shanghai, China). All aqueous solutions were prepared with de-ionized water.

### 2.2. Preparation of Magnetic Wood Fibers

The wood fibers were heated in distilled water several times until the water turned clear. Then the dried specimens were extracted with a solvent mixture of alcohol/toluene (1:2, *V/V*) overnight to remove the wood extractive compounds, such as gums, tropolones, fats, and fatty acids, and to improve the surface affinity for the iron salts. The specimens were then oven-dried at 105 °C to the moisture content of 4%. Mixture solution of FeCl_3_·6H_2_O and FeCl_2_·4H_2_O (molar ratio of Fe3^+^:Fe^2+^ = 2:1) was dissolved in distilled water to form the iron precursor solutions with a concentration of 0.45 mol/L ferric chloride. Then, the pre-treated wood fibers were impregnated in the mixture for 24 h, 48 h, and 72 h, at atmospheric pressure. After filtration and washing several times with distilled water to remove the residual iron salts on the surface, the specimens were then oven-dried at 65 °C for 12 h. After that, the dried specimens were again impregnated in a 25% ammonia solution for another 12 h. After filtration and washing several times with distilled water until reaching neutral pH, the specimens were then oven-dried at 65 °C for 24 h.

### 2.3. Preparation of Magnetic Fiber Core Board

Firstly, the prepared magnetic fibers were dried to a moisture content of 4%. Then, the isocynate adhesive was sprayed on the magnetic fibers under mechanical agitation. To be mentioned, the isocyanate adhesive accounted for 10% (wt %) of the total raw materials of the pressboard. The glued magnetic fibers were evenly deposited on a mat and pre-pressed at room temperature for 20 min with a pressure of 3 MPa. After that, the pre-pressed board was transferred to a thermocompressor for hot pressing, for which the pressure was 35 MPa at a temperature of 120 °C. The hot pressing time was controlled as 1 min per mm in thickness. Finally, the magnetic fiber core board was obtained with a density of 0.85~0.90 g/cm^3^.

### 2.4. Preparation of Magnetic Fiber Multilayer Board

**Figure 1:**
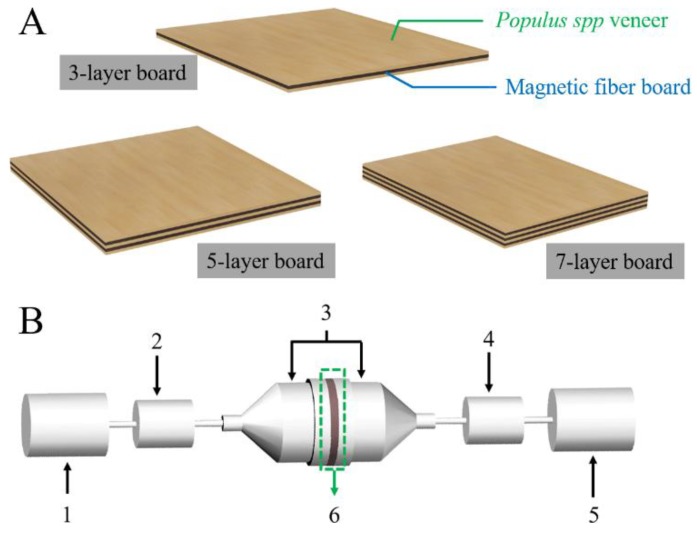
(**A**) Schematic diagram of the three different magnetic fiber multi-layer board. (**B**) The experimental components the network analyzer: 1. EMW generator; 2. attenuator; 3. specimen holder; 4. attenuator; 5. EMW receiving terminal; 6. test sample.

The dried veneer (moisture content: 6%) was manually one-side glued with isocynate adhesive (120 g/m^2^). Then, a sandwich structure was fabricated with two glued veneers and one magnetic fiber core board. The composite was hot pressed at 120 °C with a pressure of 5 MPa. The hot pressing time was controlled as 1.5 min per mm in thickness. After being cooled to room temperature in air, the three-layer magnetic composite board was obtained as diagramed in Figure 1A. By analogy, five-layer and seven-layer magnetic composite boards were prepared as diagramed in Figure 1A.

### 2.5. Characterization and Property Measurement of the as-Prepared Samples

The morphology of the magnetic fibers was characterized by scanning electron microscopy (SEM) performed using a Quanta 200 (FEI Inc., Eindhoven, The Netherlands). The EDS mapping mode was used to analyze the element distribution. XRD patterns were obtained using a Bruker D8 Advance powder X-ray diffractometer (Bruker Inc., Karlsruhe, Germany) operated at 40 kV and 40 mA using Cu-Ka radiation (λ = 1.54 Å) with 200 mg of each specimen. The data were collected with a 2θ scanning range of 10°–80°. The IR spectra were recorded by FT-IR (Nicolet 6700 FTIR spectrometer, Thermo Scientific Inc., Waltham, MA, USA), and each sample together with KBr was pressed to form a tablet. Magnetic measurements were carried out using a Lake Shore 7407 VSM (East Changing Technologies, Inc., Beijing, China).

The amount of iron oxide corresponding to different impregnation time was determined by 1,10-phenanthroline-Fe(III) spectrophotometry method. In detail, the samples were dispersed in 1L deionized water. Then we took 50 uL from the blocking solution into a 50 mL volumetric bottle and added 2 mL HCl solution (6 M). The mixture was treated by ultrasound for 30 min, followed by in-turn addition of 1 mL NH_4_OH·HCl (10 %) solution, 2 mL 1,10-phenanthroline (0.1%) solution, 2 mL NaOH (6 M) solution, and 5 mL NaAc-HAc buffer (pH = 5.0). Finally, the mixed solution was fixed to 50 mL, and the corresponding absorbance at 510 nm was measured by UV spectrophotometer, which is directly proportional to the amount of iron in magnetic wood fiber.

For the EMW absorbing property measurement, coaxial-line method was used based on the fact that coaxial-line method needs a smaller amount of the sample and it can obtain the scattering parameter (S parameter) along with the corresponding complex permittivity and permeability parameters of the samples, which are very important for the further investigation of the corresponding EMW absorbing mechanism. The specimens were firstly manufactured into a torodial-shape (Φout: 7.0 mm, Φin: 3.04 mm) by a special electric motor (Type: SM4955B-18/2.2TMP, Rund Awords^®^). Afterwards, the relative complex permittivities and relative complex permeabilities of the specimens were measured using an Agilent N5230C network analyzer in the frequency range of 2–18 GHz. The experimental components of the device along with the test sample is shown in Figure 1B. To be mentioned, based on the consideration of usual using habits, the specimens were manufactured perpendicular to the plane.

The EMW absorbing performance of the specimens can be evaluated by the reflection loss (RL), which can be defined with the following equations on the basis of transmission line theory [23]:

(1) $$Z_{in}=Z_{0}{\left(\mu_{r}/\varepsilon_{r}\right)}^{1/2}\tan h[j\left(2\pi fd/c\right){\left(\mu_{r}\varepsilon_{r}\right)}^{1/2}]$$

(2) $$RL=20\log_{10}\left|\left(Z_{in}-Z_{0}\right)/\left(Z_{in}+Z_{0}\right)\right|$$

where *Z_0_* is the characteristic impedance of free space, *Z_in_* is the input impedance of the absorber, *ε_r_* is the relative complex permittivity ($\varepsilon_{r}=\varepsilon^{\prime }-j\varepsilon^{\prime \prime }$), *μ_r_* is the relative complex permeability ($\mu_{r}=\mu^{\prime }-j\mu^{\prime \prime }$), *f* is the frequency of the microwaves, *d* is the thickness of the specimen, and *c* is the velocity of light. To be mentioned, all the results (including $\varepsilon^{\prime }$, $\varepsilon^{\prime \prime }$, $\mu^{\prime }$, $\mu^{\prime \prime }$) were tested and collected as average values from the samples obtained by three independently repeated experiments. And all the corresponding EMW absorbing capacities, along with the *C_0_* values and the tangent values were calculated based on these average values.

## 3. Results and Discussion

### 3.1. Morphological and Component Characterization of Magnetic Wood Fibers

**Figure 2:**
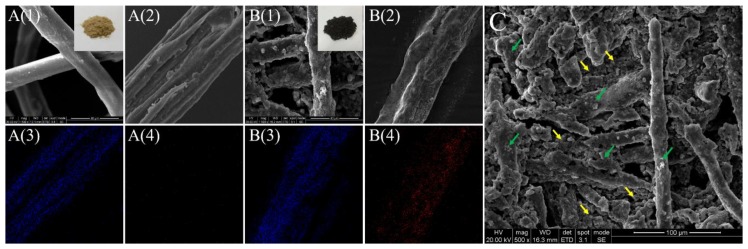
SEM and EDS images of the (**A**) untreated and (**B**) treated wood fibers. (3), (4) are EDS mappings of O and Fe for the separate wood fibers in **A**(2) and **B**(2), respectively. Insert: macro-structures of two samples. (**C**) is the SEM image of treated wood fibers in a large view. The typical particles and clusters in the spaces among the fibers and on the fiber surface are pointed to with yellow and green arrows, respectively.

Figure 2 shows the SEM observations of the untreated (Figure 2A) and treated (Figure 2B) wood fibers. The inserted images are the macro-structures of two samples. It is obvious that the fibers with impregnation treatments were dark brown compared with the untreated ones. This color change is attributed to the presence of Fe_3_O_4_ nanoparticles that might be generated during the treatments. Besides, compared with the untreated wood fibers, the treated wood fibers obviously illustrated rougher surfaces, covered continuously by clusters of particles. Confirmed by EDS mapping images in Figure 2B(4), the intensity and density of Fe signals were higher for the treated wood fiber compared to the natural wood fiber, implying that these attached particles and clusters consisted of iron elements. The mass ratio of the contained Fe element in the magnetic wood fibers obtained with different impregnation time, which corresponds to the amount of the attached particles, are listed in Table 1. As shown in Table 1, the content of Fe element in the magnetic fiber increased with the impregnation time, indicating more formation of Fe_3_O_4_ attaching on the wood fiber surface. To be mentioned, the attached clusters of the particles on the surface of the wood fibers were observed to be loosely structured, which was beneficial to the penetration of isocyanate adhesives to these surfaces, and to achieve the adhesive and hot-pressing processes. Besides, according to Figure 2C, we observed that the formed particles were present both in the spaces among the fibers and on the fiber surface, in the form of clusters with micrometer-size.

**Figure 3:**
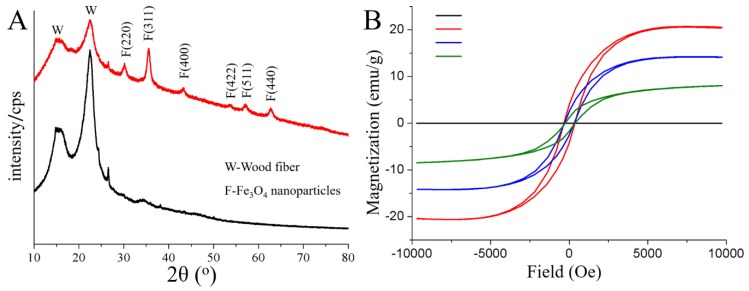
(**A**) XRD patterns of the untreated (black) and treated (red) wood fibers. (**B**) VSM curves of the untreated wood fibers (black), and magnetic wood fibers with impregnation time of 24 h (green), 48 h (blue), and 72 h (red), respectively.

To confirm the chemical compositions of the treated wood fibers, the crystalline structures and phase compositions of the treat and untreated wood fibers were studied by wide-angle XRD as shown in Figure 3A. We noticed that both of the samples displayed two primary diffraction peaks at 16.0° and 22.5°, which can be assigned to the (100) and (002) planes of cellulose, respectively [24]. The treated wood fibers possessed additional diffraction peaks at 2θ = 30.0, 35.3, 43.0, 53.4, 56.9, and 62.5, corresponding to the (220), (311), (400), (422), (511), and (440) planes of Fe_3_O_4_ in a cubic phase, respectively [25]. According to the Scherrer equation:

(3) d=Kλ/βcosθ,

where *d* is the mean diameter of the Fe_3_O_4_ nanoparticles, *K* is a dimensionless shape factor (0.89), *λ* is the X-ray wavelength (0.154 nm), *β* is the line broadening at half the maximum intensity corresponding to the most intense peak (311), and θ is the Bragg angle; the average crystallite size was found to be approximately 21.8 nm, indicating that the attached clusters obtained in SEM results are formed by Fe_3_O_4_ particles in nano-size.

### 3.2. Magnetic Properties of the Magnetic Wood Fibers

The magnetic properties of the prepared magnetic wood fibers were measured by VSM. As seen in Figure 3B, the typical characteristics of magnetic behavior for the treated and untreated wood fibers are observed. It is obvious that the saturation magnetization values ($M_{s}$) were 8.04, 14.10, and 20.38 emu/g for the samples obtained via 24 h-, 48 h- and 72 h-impregnation, respectively. This increasing tendency in $M_{s}$ value along with the increase of iron salt immersion time, is attributed to the increasing of in-situ generated Fe_3_O_4_ attachment on the wood fiber surface (as shown in Table 1). Additionally, the observed curves also showed that all the samples exhibited a clear hysteretic behavior. The intercept of the hysteresis loop on the X-axis represents the magnetic coercive force ($H_{c}$) of the magnetic wood fibers, and the corresponding value reflects their ability to retain the remanent state, according to which magnetic materials are generally divided into hard and soft magnetic materials. From Figure 3B, we may see that the as-prepared magnetic wood fibers, compounding of Fe_3_O_4_ clusters and natural *Populus* spp. fibers, possessed a large $H_{c}$ value (~315 Oe), which was dramatically different from the superparamagnetic behavior of Fe_3_O_4_ in nano-size. This may be due to the different dispersity of the magnetic component. According to Stoner-Wohlfarth theory [26], *Ms* is related to the magnetocrystalline anisotropy (*K_eff_*) of the specimen:

(4) $$K_{eff}=M_{S}\mu_{0}H_{C}/2,$$

where *μ*_0_ is a constant of permeability. Thus, the increase of *M_S_* values among the specimens together with the large $H_{c}$ value should be interpreted as an increase in *K_eff_* with the increasing of Fe_3_O_4_ attachment. Surface anisotropy (*K_s_*), which is another relevant parameter to coercivity and contributes to the effective *K_eff_* [27]:

(5) $$K_{eff}=K_{b}+\left(6/d\right)K_{s},$$

where *K_b_* is the bulk anisotropy and was probably not changed in this work. *d* is the diameter of the Fe_3_O_4_ nanoparticles. In this equation, we may see that *K_s_* is positively correlated with *K_eff_*, indicating that *K_s_* also increase with the increasing of the Fe_3_O_4_ attachment. However, *K_s_* is supposed to be at its maximum for the free surfaces and is reduced by solid coverage. This unusual increasing of *K_s_* should be attributed to the fact that more Fe_3_O_4_ nanoparticles aggregated in the form of generated clusters on the wood fibers, inducing a rougher exterior surface and further affecting the contribution of the surface anisotropy (*K_s_*) (as shown in Figure 2B(1)). In fact, this anisotropic exterior surface was conducive to the corresponding EMW absorption properties.

### 3.3. EMW Absorbing Properties of Magnetic Fiber Board

**Figure 4:**
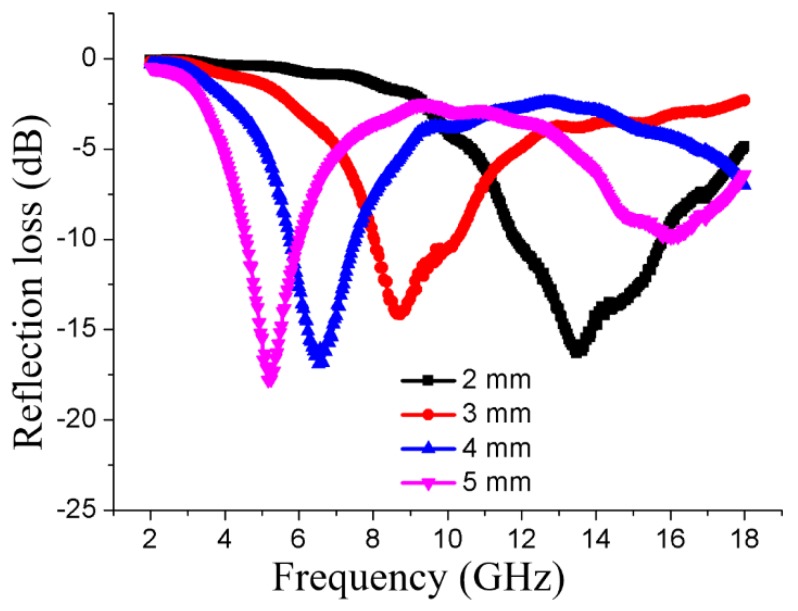
The reflection loss curves of the 3-layer composite magnetic board fabricated by a magnetic fiber core with the thickness from 2 mm to 5 mm.

Figure 4 shows the calculated reflection-loss properties of the magnetic fiber boards with different thickness, which were obtained after 24 h impregnation of the iron solution. As can be seen, the magnetic fiber board of varied thickness of 2 mm, 3 mm, 4 mm, and 5 mm, possessed similar EMW absorbing capacities, which were −16.31 dB, −14.14 dB, −16.91 dB, and −17.81 dB with a decreasing matching frequency at 13.48 GHz, 8.64 GHz, 6.52 GHz, and 5.16 GHz, respectively. As we know, the |RL|>10 dB means 90% attenuation of electromagnetic wave, which has been an effective *RL* value. And an ideal EMW absorber is required to have not only a strong absorption, but also a wide absorbing bandwidth, where corresponding |RL| is larger than 10 dB. The effective absorbing bandwidths of all these thickness were 3.88 GHz (from 11.88 GHz to 15.76 GHz), 2.16 GHz (from 8.04 GHz to 10.20 GHz), 1.72 GHz (from 5.76 GHz to 7.48 GHz), and 1.40 GHz (from 4.60 GHz to 6.00 GHz), respectively. Therefore, we can conclude that, with the increase of the thickness of the magnetic fiber core, the EMW absorbing properties of the fabricated composite magnetic board changed little, while the corresponding matching frequency and effective absorbing frequency range decreased. This phenomenon can be explained by the 1/4 wavelength cancelation law [28]:

(6) $$t_{m}=nc/4f_{m}{\left(\varepsilon_{r}\mu_{r}\right)}^{1/2}\left(n=1,3,5,\ldots \right),$$

where $t_{m}$ is the thickness of the core material of the magnetic fiber board, and $f_{m}$ is the electromagnetic wave frequency corresponding to the maximum shielding intensity of the electromagnetic wave. When $t_{m}$ and $f_{m}$ satisfy this equation, the reflected electromagnetic microwaves both from the air-absorber interface and the absorber-conductive background interface are out of phase by 180°, inducing an extinction of them on the air-absorber interface, and then resulting in a maximum *RL* value.

**Figure 5:**
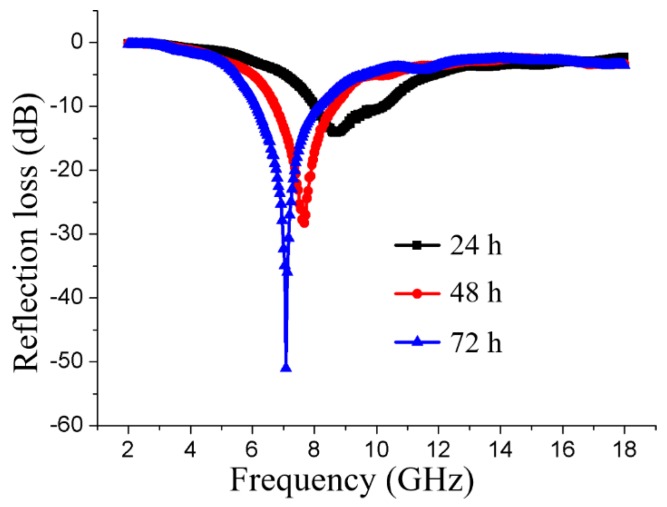
The reflection loss curves of the three-layer composite magnetic board fabricated by a magnetic fiber core with the thickness of 3 mm. The impregnation time was 24 h, 48 h, and 72 h, respectively.

To investigate the influence of the iron salt impregnation time on the EMW absorbing properties of the magnetic fiber board, the EMW absorbing performance of the magnetic fiber board with a thickness of 3 mm was tested. Here, the magnetic fiber board was fabricated with the magnetic wood fibers which were obtained through different iron salt impregnation time of 24 h, 48 h, and 72 h respectively. Figure 5 shows the calculated reflection-loss properties of the specimens in the frequency range of 2–18 GHz. As shown in Figure 5, the immersion time had dramatic effects on the EMW absorbing capacities of the specimens. When the thickness was set as 3 mm, the specimen with 24 h impregnation time exhibits a weaker absorption capacity of −14.14 dB at 8.64 GHz, while the specimens with 48 h and 72 h impregnation time exhibited stronger adsorption capacities of −28.28 dB and −51.01 dB, respectively. These results indicated that, the EMW absorbing properties were enhanced with the increase of the iron salt impregnation time, this may be related to the deposition of the magnetic particles on the wood fiber surface.

**Figure 6:**
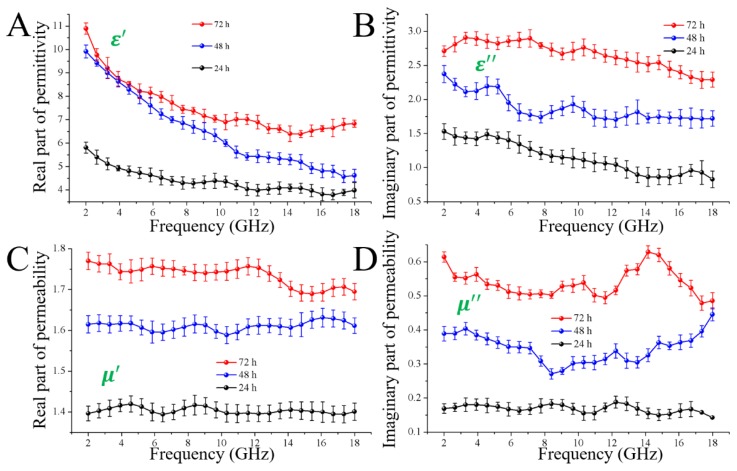
Frequency dependences of real parts (**A**) and imaginary parts (**B**) of complex permittivities, and the real parts (**C**) and imaginary parts (**D**) of complex permeabilities of the magnetic fiber boards with different impregnation time, 24 h, 48 h and 72 h, respectively.

Based on Equations (1) and (2), the EMW absorbing properties of the specimens were dependent on the relative complex permittivity and the relative complex permeability. To investigate the origin of the varied EMW absorption performances of the specimens obtained through different impregnation time, the complex permittivity, and permeability were investigated over the frequency range of 2.0–18.0 GHz. Figure 6A,B show the real part (ε′) and the imaginary part (ε′′) of permittivity, which represent the storage capability and the loss capability of the electric, respectively [29]. As shown in Figure 6A,B, the specimen with 72 h impregnation time exhibited the highest ε′ value (ranging from 10.89 to 6.39) and the largest ε′′ value (ranging from 2.91 to 2.29), indicating the conductivities of the samples gradually increased with the increase of the impregnation time from 24 h to 72 h. This induced not only the good impedance matching behavior, but also a strong ability for the electromagnetic attenuation of the specimen obtained with 72 h impregnation. Moreover, the ε′ values of all the magnetic fiber boards gradually decreased with the increasing frequency, which is attributed to the fact that the lagging of polarization increases with respect to the electric-field change at high frequency [30]. Several resonant peaks were observed in the ε′′ curves, which play a key role on the attenuation of the electromagnetic wave. For the microwave frequency (GHz), these peaks are mainly attributed to the multiple polarization relaxation processes occurring in the magnetic fiber board under alternating electromagnetic field [31]. As we know, the exterior surface of the natural wood fibers is functioned with oxygen-containing chemical bonds, such as –OH and –CO–, which produce electronic dipolar polarization. Meanwhile, the peaks were also induced by the interfacial polarization, coming mainly from the interface between Fe_3_O_4_ clusters and the carbohydrates from the wood fibers. As being supposed, both interface polarization and dipole relaxation polarization were positive to improve the microwave absorption performance.

Figure 6C,D reveals that both $\mu^{\prime }$ and $\mu^{\prime \prime }$ values of the specimen obtained with 72 h impregnation were dramatically higher than the other samples at all the tested frequencies, which should induce the best impedance-matching behavior. According to the following equations [32]:

(7) $$\mu^{\prime }=1+\left(\frac{M}{H}\right)\cos\sigma ;$$

(8) $$\mu^{\prime \prime }=1+\left(\frac{M}{H}\right)\sin\sigma ,$$

where *M* is the magnetization, *H* is the intensity of the external magnetic field, and *σ* is the phase lag angle, the increased trend of the permeability is attributed to the increasing of saturation magnetization of the magnetic wood. In addition, several resonant peaks can be observed in the $\mu^{\prime \prime }$ curves of nearly all the samples. In general, the formation of these peaks are attributed to exchange resonance, eddy current resonance, and natural resonances [33]. As we know, eddy current resonance is needed to be suppressed but hardly avoided in a design of EMW absorbing materials, since it is supposed to prevent the EMW from coming into the absorber. According to the criterion of skin effect, the effect of the eddy currents on the magnetic loss is evaluated by analyzing the variation trend of $C_{0}$ values based on the following equation [34]:

(9) $$C_{0}=\mu^{\prime \prime }{\left(\mu^{\prime }\right)}^{-2}f^{-1}$$

**Figure 7:**
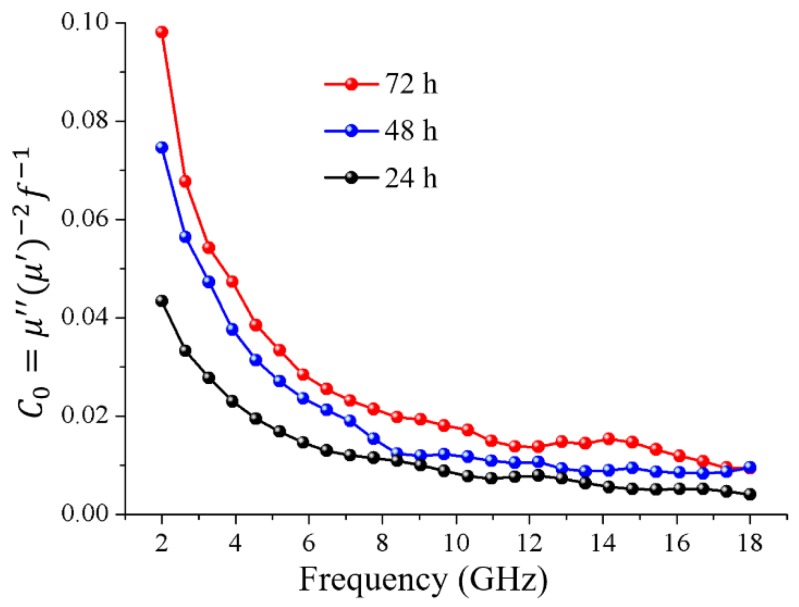
Plots of $\mu^{\prime \prime }{\left(\mu^{\prime }\right)}^{-2}f^{-1}$ vs. frequency for all the as-prepared magnetic wood fibers.

Equation (9) implies that the plots of $C_{0}$ remain stable as a constant with the changing frequency, in the case of magnetic loss only being contributed to the eddy current loss. As shown in Figure 7, the $C_{0}$ values vary with frequency, indicating that the eddy current effect could be excluded, and the natural resonance and exchange resonance may be the main contributors to the magnetic loss.

**Figure 8:**
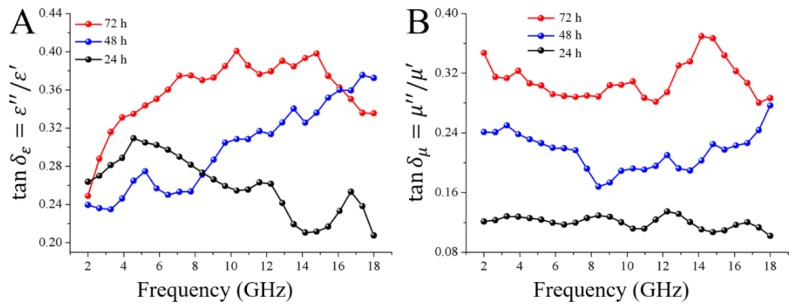
Frequency dependences of $\tan\delta_{\varepsilon }$ and $\tan\delta_{\mu }$ of magnetic wood: 24 h (black), 48 h (blue), and 72 h (red).

The dielectric-loss tangent ($\tan\delta_{\varepsilon }=\varepsilon^{\prime \prime }/\varepsilon^{\prime }$) and the magnetic loss tangent ($\tan\delta_{\mu }=\mu^{\prime \prime }/\mu^{\prime }$) are usually used to estimate the loss ability of the microwave [35]. As shown in Figure 8A, it is obvious that the $\tan\delta_{\varepsilon }$ values increased with the impregnation time from 24 h to 72 h. The specimen obtained with 72 h impregnation time possessed the highest $\tan\delta_{\varepsilon }$ value of 0.40 at 10.32 GHz, implying its highest capacity of converting the EMW to the energy in other forms. This was very important to the electromagnetic wave absorbing properties. According to the SEM results in Figure 2, the higher loss tangent in terms of permittivity is attributed to the larger quantity of Fe_3_O_4_ nanoparticles as the conductive component in sample, which covered the entire wood fiber surface. And during the hot-pressing processes, the fabricated magnetic wood fibers formed an interconnected conductive network spreading over the magnetic fiber board for electron hopping and migrating. The magnetic loss tangent was also investigated in Figure 8B. As shown in Figure 8B, the specimen obtained with 72 h impregnation time possesses the highest $\tan\delta_{\mu }$ value of 0.37 at 14.16 GHz. This value was close to the $\tan\delta_{\varepsilon }$ values, suggesting that both the dielectric loss and the magnetic loss played the dominant roles in all the samples and the resulted different RL capacities were determined by the matching degree between dielectric loss tangent and magnetic loss tangent.

**Figure 9:**
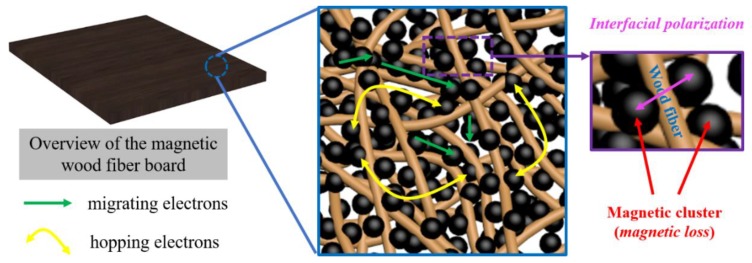
Scheme of primary EMI absorbing processes in the magnetic fiber board (Left): interfacial polarization (Right) at the interfaces between Fe_3_O_4_ nanoparticles and the carbohydrates from the wood fiber surface, and conductive network (Middle) constructed by magnetic nanoparticles. The nanoparticles attached on the wood fiber surface and in the spaces among these fibers (as observed in Figure 3C) are labeled as black.

In conclusion, the magnetic fiber board obtained with the iron salt impregnation time of 72 h, possesses the optimal impedance matching. The EMW energy was converted to heat energy in the magnetic board. The strongest dielectric loss was caused by the interconnected conductive network spreading over the magnetic fiber board for electron hopping and migrating, which was formed by the wood fiber framework modified with Fe_3_O_4_ nanoparticles, and by the interfacial polarization between the magnetic clusters and the carbohydrates from the wood fibers. The optimal magnetic loss was caused by natural resonance and exchange resonance. Additionally, the dipole relaxation polarization which was brought out by the enriched oxygen-containing chemical groups, was positive to improve the microwave absorption performance (as shown in Figure 9).

### 3.4. EMW Absorbing Properties of the Magnetic Composite Multi-Layer Board

**Figure 10:**
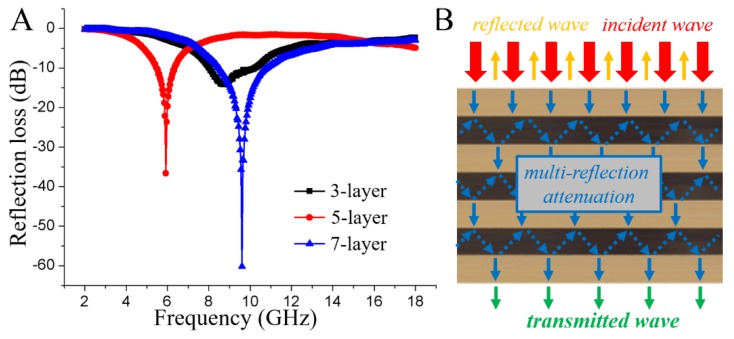
(**A**) The reflection loss curves of 3-, 5-, and 7-layer magnetic composite boards. (**B**) Schematic illustrating the EMW absorbing mechanisms for multi-layer magnetic composite boards perpendicular to the plane.

As shown in Figure 10A, EMW absorption performance of 3-, 5- and 7-layer magnetic composite boards with the magnetic fiber board core prepared with 24 h impregnation time with the thickness of 3 mm (as shown in Figure 1A). From Figure10A we may see that the EMW absorption capacities increased from −14.14 dB (3-layer) to −60.16 dB (7-layer) with the increase of the number of magnetic fiber board from one to three, respectively. The alignment of the magnetic fiber boards along the axial direction generated multi-interfaces between magnetic fiber board and natural wood veneer in the EMW propagation direction, significantly benefiting multireflection and attenuation of the incident waves (Figure 10B).

## 4. Conclusions

In summary, we developed a facile in situ chemical co-precipitation method coupled with hot-pressing processes to fabricate magnetic composite multi-layer board as EMW absorbing materials. The EMW absorbing properties of the as-prepared magnetic fiber boards could be adjusted via the iron salt impregnation time and the thickness. The specimens obtained 72 h impregnation time exhibited strongest adsorption capacities of −51.01 dB with a thickness of 3.00 mm. This outstanding EMI absorption property was proved to be due to the corresponding strongest dielectric loss, optimal magnetic loss, and dipole relaxation polarization. Meanwhile, the EMW absorbing capacities of the corresponding multi-layer composite magnetic board could be adjusted via the number of the consisting magnetic fiber boards, which increased from −14.14 dB (3-layer board containing one magnetic fiber board) to −60.16 dB (7-layer magnetic board containing three magnetic fiber boards). This is due to the generated multi-interfaces between magnetic fiber board and natural wood veneer in the EMW propagation direction, which significantly benefited multireflection and attenuation of the incident waves.

## Figures and Tables

**Table 1:** The mass ratio of Fe element in the magnetic wood fiber obtained in different impregnation time.

**Impregnation Time**	24 h	48 h	72 h
**Mass Ratio (%) of Fe**	10.3 ± 1.2	27.0 ± 1.8	33.0 ± 1.5
